# Supplementary material for: The Potential Applications of Commercial Arbuscular Mycorrhizal Fungal Inoculants and Their Ecological Consequences
Source: Microorganisms. 2022 Sep 23;10(10):1897. doi: 10.3390/microorganisms10101897 (PMC9609176; doi:10.3390/microorganisms10101897)
Supplement: Supplementary file 1 [file microorganisms-10-01897-s001.zip › microorganisms-1899255-supplementary.pdf]

Figure S1: Country location of studies investigating ecological fates of AMF inoculants.

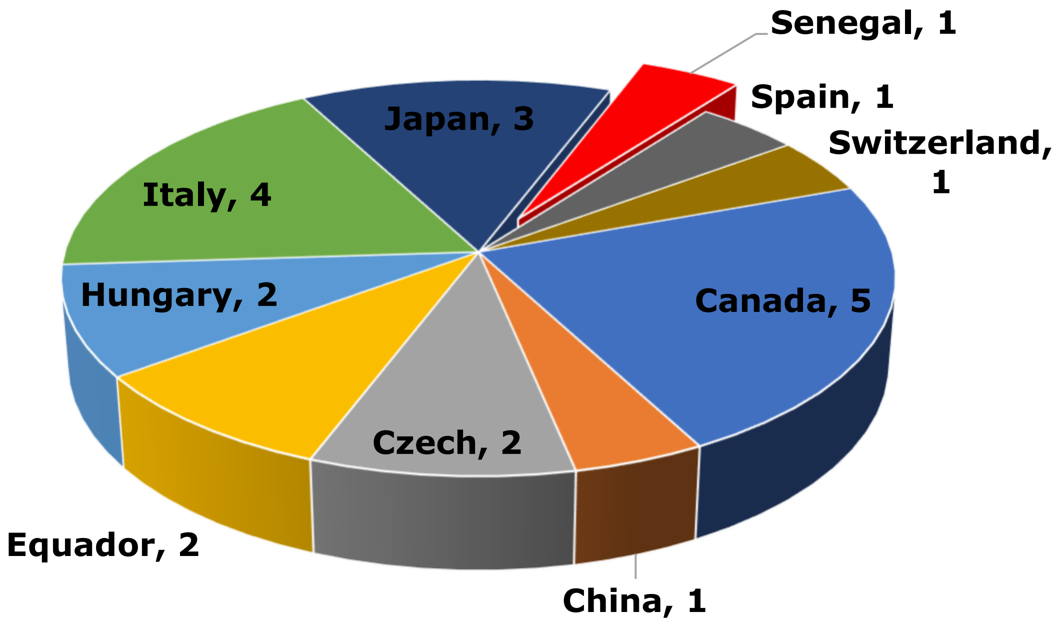

Figure S2: Search results on effect of AMF inoculants on indigenous AMF in the field indicating: A) source of inoculum; B) site-specific effects on composition and structure of indigenous AMF (Yes means observed effect; No means no effect).

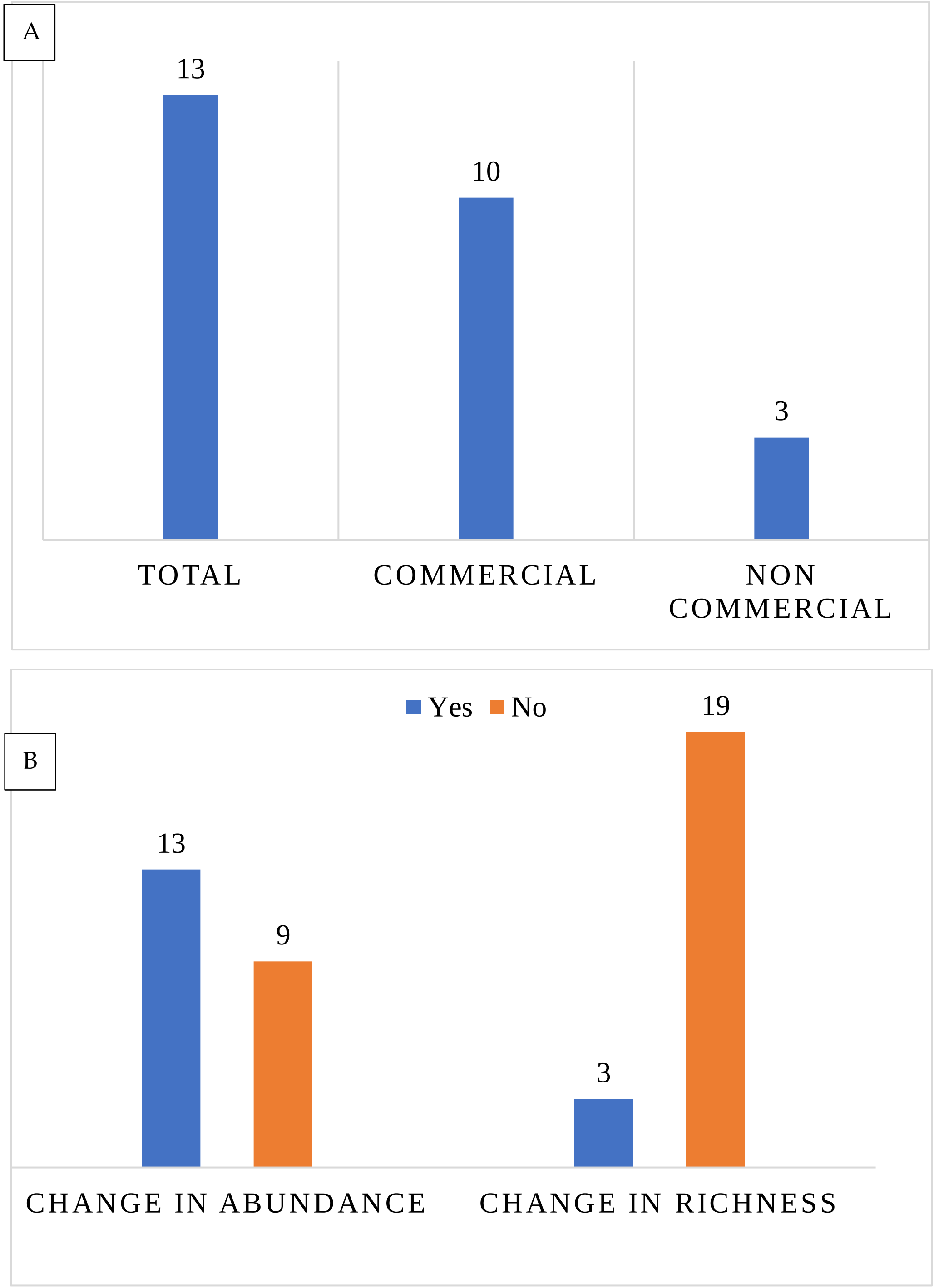

List of studies filtered according to the criteria described in the article.

Akyol TY, Niwa R, Hirakawa H, Maruyama H, Sato T, Suzuki T, et al. Impact of Introduction of Arbuscular Mycorrhizal Fungi on the Root Microbial Community in Agricultural Fields. Microbes Environ. 2019;34(1):23-32.

Bender SF, Schlaeppi K, Held A, Van der Heijden MGA. Establishment success and crop growth effects of an arbuscular mycorrhizal fungus inoculated into Swiss corn fields. 2019.

Berruti A, Lumini E, Bianciotto V. AMF components from a microbial inoculum fail to colonize roots and lack soil persistence in an arable maize field. Symbiosis. 2017;72(1):73-80.

Hernádi I, Magurno F, Sasvári Z, Posta K. Effects of mycorrhizal inoculants on cultivation of two spice pepper types and local arbuscular mycorrhizal fungal community. 2017.

Hernádi I, Sasvári Z, Albrechtová J, Vosátka M, Posta K. Arbuscular mycorrhizal inoculant increases yield of spice pepper and affects the indigenous fungal community in the field. 2012;2012.

Islam MN, Germida JJ, Walley FL. Survival of a commercial AM fungal inoculant and its impact on indigenous AM fungal communities in field soils. Applied Soil Ecology. 2021;166.

Li Y, Gan Y, Lupwayi N, Hamel C. Influence of introduced arbuscular mycorrhizal fungi and phosphorus sources on plant traits, soil properties, and rhizosphere microbial communities in organic legume-flax rotation. Plant and Soil. 2019;443(1-2):87-106.

Li Y, Laterrière M, Lay C-Y, Klabi R, Masse J, St-Arnaud M, et al. Effects of arbuscular mycorrhizal fungi inoculation and crop sequence on root-associated microbiome, crop productivity and nutrient uptake in wheat-based and flax-based cropping systems. Applied Soil Ecology. 2021;168.

Loján P, Senés-Guerrero C, Suárez JP, Kromann P, Schüßler A, Declerck S. Potato field-inoculation in Ecuador with Rhizophagus irregularis: no impact on growth performance and associated arbuscular mycorrhizal fungal communities. Symbiosis. 2016;73(1):45-56.

Kokkoris V, Li Y, Hamel C, Hanson K, Hart M. Site specificity in establishment of a commercial arbuscular mycorrhizal fungal inoculant. Sci Total Environ. 2019;660:1135-43.Niwa R, Koyama T, Sato T, Adachi K, Tawaraya K, Sato S, et al. Dissection of niche competition between introduced and indigenous arbuscular mycorrhizal fungi with respect to soybean yield responses. 2018.

Pellegrino E, Nuti M, Ercoli L. Multiple Arbuscular Mycorrhizal Fungal Consortia Enhance Yield and Fatty Acids of Medicago sativa: A Two-Year Field Study on Agronomic Traits and Tracing of Fungal Persistence. Front Plant Sci. 2022;13:814401.

Renaut S, Daoud R, Masse J, Vialle A, Hijri M. Inoculation with Rhizophagus Irregularis Does Not Alter Arbuscular Mycorrhizal Fungal Community Structure within the Roots of Corn, Wheat, and Soybean Crops. Microorg. 2020;8(1).

Thioye B, Sanguin H, Kane A, Ndiaye C, Fall D, Sanogo D, et al. Mycorrhizal inoculation increases fruit production without disturbance of native arbuscular mycorrhizal community in jujube tree orchards (Senegal). Symbiosis. 2021;83(3):361-72.

Victorino IMM, Voyron S, Caser M, Orgiazzi A, Demasi S, Berruti A, et al. Metabarcoding of Soil Fungal Communities Associated with Alpine Field-Grown Saffron (Crocus sativus L.) Inoculated with AM Fungi. J Fungi (Basel). 2021;7(1).
